# Supplementary material for: YAP1 subgroup supratentorial ependymoma requires TEAD and nuclear factor I-mediated transcriptional programmes for tumorigenesis
Source: Nat Commun. 2019 Sep 2;10:3914. doi: 10.1038/s41467-019-11884-5 (PMC6718408; doi:10.1038/s41467-019-11884-5)
Supplement: Supplementary file 1 — Supplementary Information [file 41467_2019_11884_MOESM1_ESM.pdf]

SUPPLEMENTARY INFORMATION

YAP1 subgroup supratentorial ependymoma requires TEAD and Nuclear factor I-mediated transcriptional programmes for tumourigenesis

Pajtler et al.

Supplementary Figure 1-9

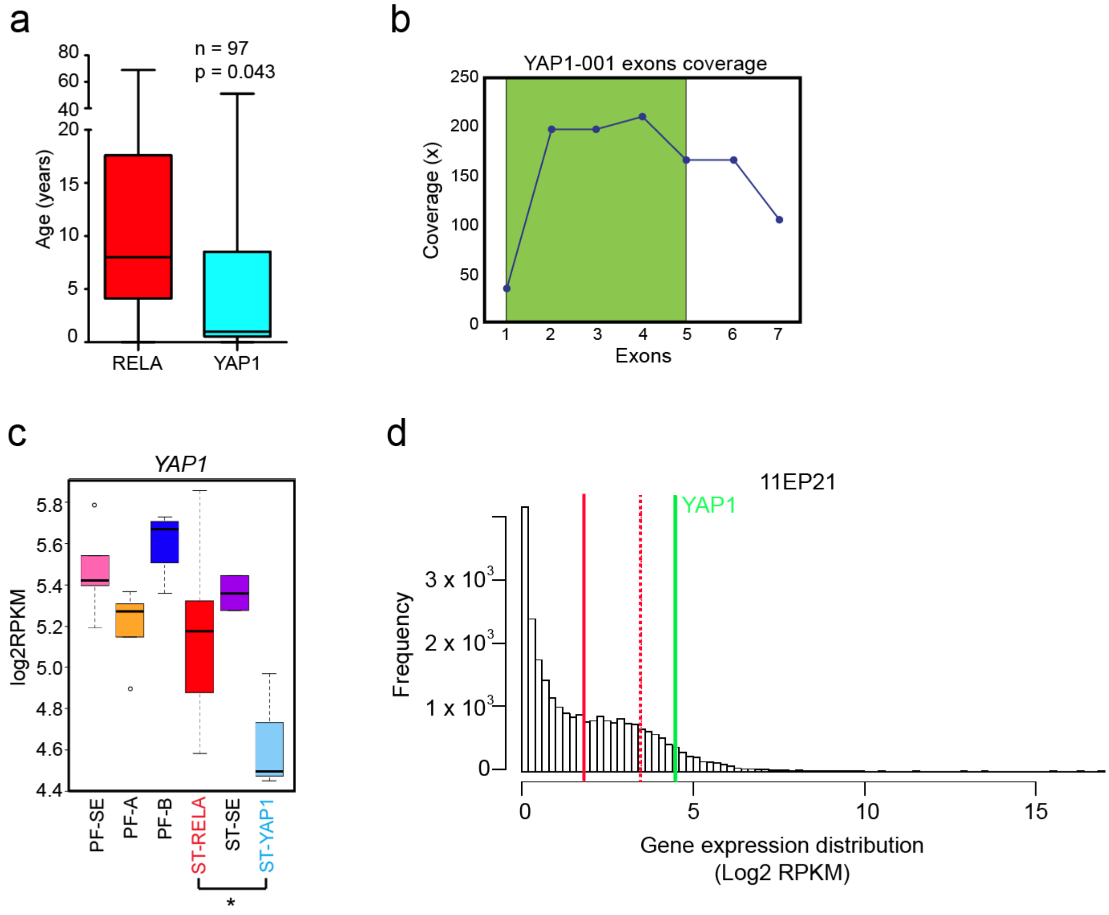

Supplementary Figure 1:

YAP1 mRNA expression levels in different molecular ependymoma groups.

(a) Box plots for age of patient at tumour onset. Statistical significance of age was determined by a Mann-Whitney U test. The centre line, box limits and whiskers indicate the median, upper/lower quartiles and 1.5x interquartile range respectively.

(b) Split read data from RNA-seq generated from a ST-EPN-YAP1 specimen (YAP1#1). Green area (Exon 1-5) represents the region of the *YAP1* gene encoded by both YAP1 wildtype and the fusion gene. In this sample, 23 fusion split reads and 76 normal gene reads were detected, thus indicating 23.2% of the YAP1 transcripts are from the YAP1 fusion gene.

(c) Box plots of YAP1 mRNA expression in different molecular ependymoma groups based on RNA sequencing data [5]. *p-value = 0.2589 (adjustment computed by Benjamini & Hochberg method). The centre line, box limits, whiskers and points indicate the median, upper/lower quartiles, 1.5x interquartile range and outliers respectively. Of note, the value for ST-YAP1 group includes both YAP1 wildtype and YAP1 fusion genes.

(d) Gene expression distribution in a human ST-EPN-YAP1#3 (11EP21). The red lines reflect mean and standard deviation (dotted), green line marks YAP1 expression level.

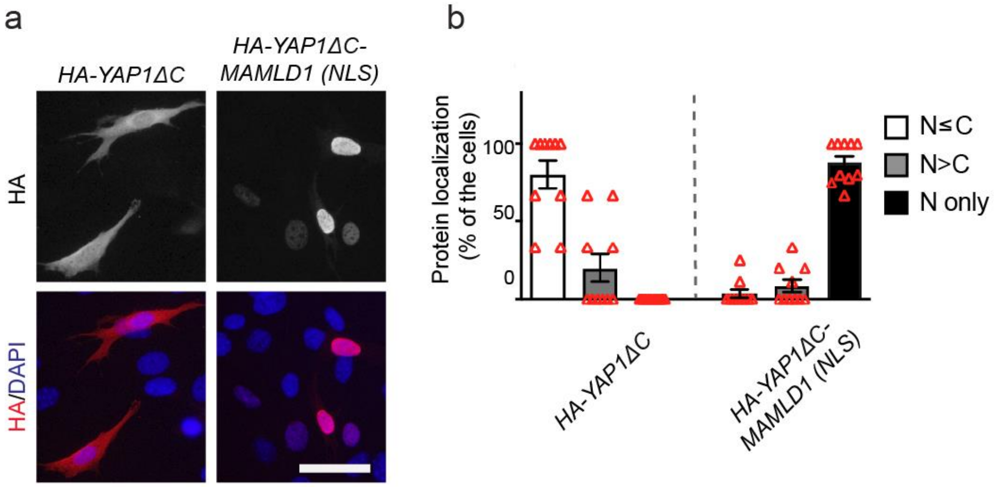

Supplementary Figure 2:

The MAMLD1 domain induces nuclear localization of the YAP1 protein.

(a) NIH/3T3 cells transiently transfected by indicated genes tagged by HA were subjected to immunofluorescent staining as indicated. Scale bar, 50 µm.

(b) The quantified results of the relative amount of each HA-tagged protein in cell nucleus (N) and cytoplasm (C) from the immunofluorescent staining in (a). The error bars indicate mean ± S.D. (n = 10).

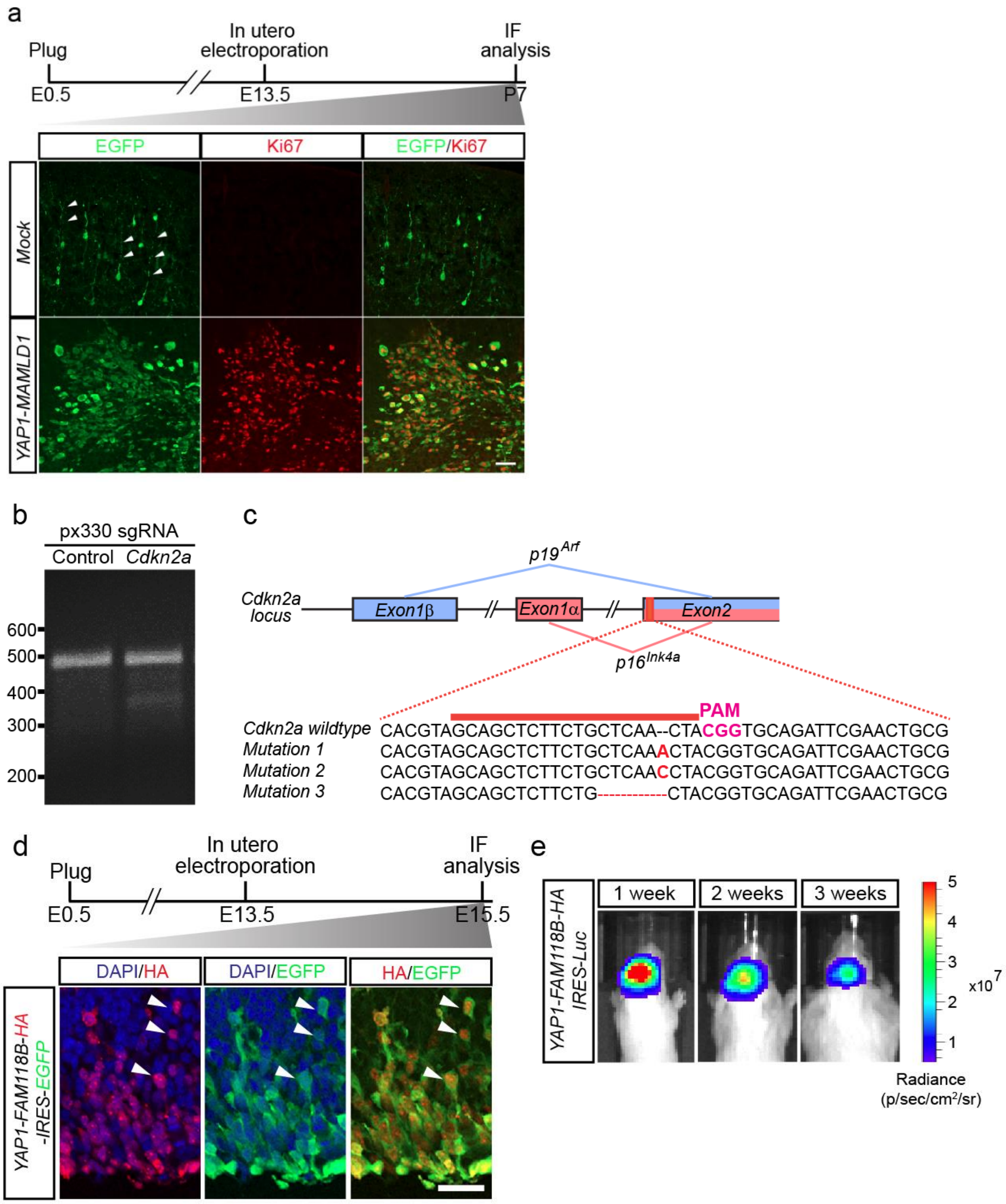

Supplementary Figure 3:

Analyses of electroporation-based in vivo mouse ST-EPN modelling.

(a) Morphologies of neural progenitors that were electroporated at E13.5 with *T2TP* and either *EGFP* (upper panels) or *YAP1-MAMLD1-IRES-EGFP*. P7 cortical plate immunostained with anti-GFP (green) and Ki67 (red) antibodies. Arrowheads indicate neurites of electroporated cells. Scale bar, 50 μm.

(b) Surveyor assay for the sgRNA targeting the *Cdkn2a* loci: GCAGCTCTTCTGCTCAACTA. As a negative control, the following sequence that does not map to the mm10 mouse genome assembly, even when changing any two positions, was used (Zuckermann et al., 2015): Control, GCGACCAATACGCGAACGTC.

The upper bands (500 bp) indicated unmodified PCR products and the lower bands (320 + 180 bp) were PCR products cleaved by S1 endonucleases. The calculated indel ratio was ~7%.

(c) Indels detected in tumours induced by electroporation of *C11orf95-RELA* and the sgRNA targeting *Cdkn2a*. Red line indicates the targeted sgRNA region in the exon 2 of *Cdkn2a*. PAM = protospacer adjacent motif.

(d) Immunofluorescence micrographs for subcellular localization of the YAP1-FAM118B-HA protein in the cells of the ventricular zone 2 days after in utero electroporation of pT2K YAP1-FAM118B-HA-IRES-EGFP plasmid at E13.5. Double staining was performed for DAPI/HA (left panel) or DAPI/EGFP (mid panel) or EGFP/HA (right panel). Arrowheads indicate nuclear localization of YAP1-FAM118B protein in electroporated cells. Scale bar, 20 µm.

(e) Luciferase-based in vivo bioluminescence images of a postnatal mouse subjected to electroporation of *YAP1-FAM118B-HA-IRES-Luc* at E13.5. The luciferase signals largely disappeared by one month after birth (data not shown).

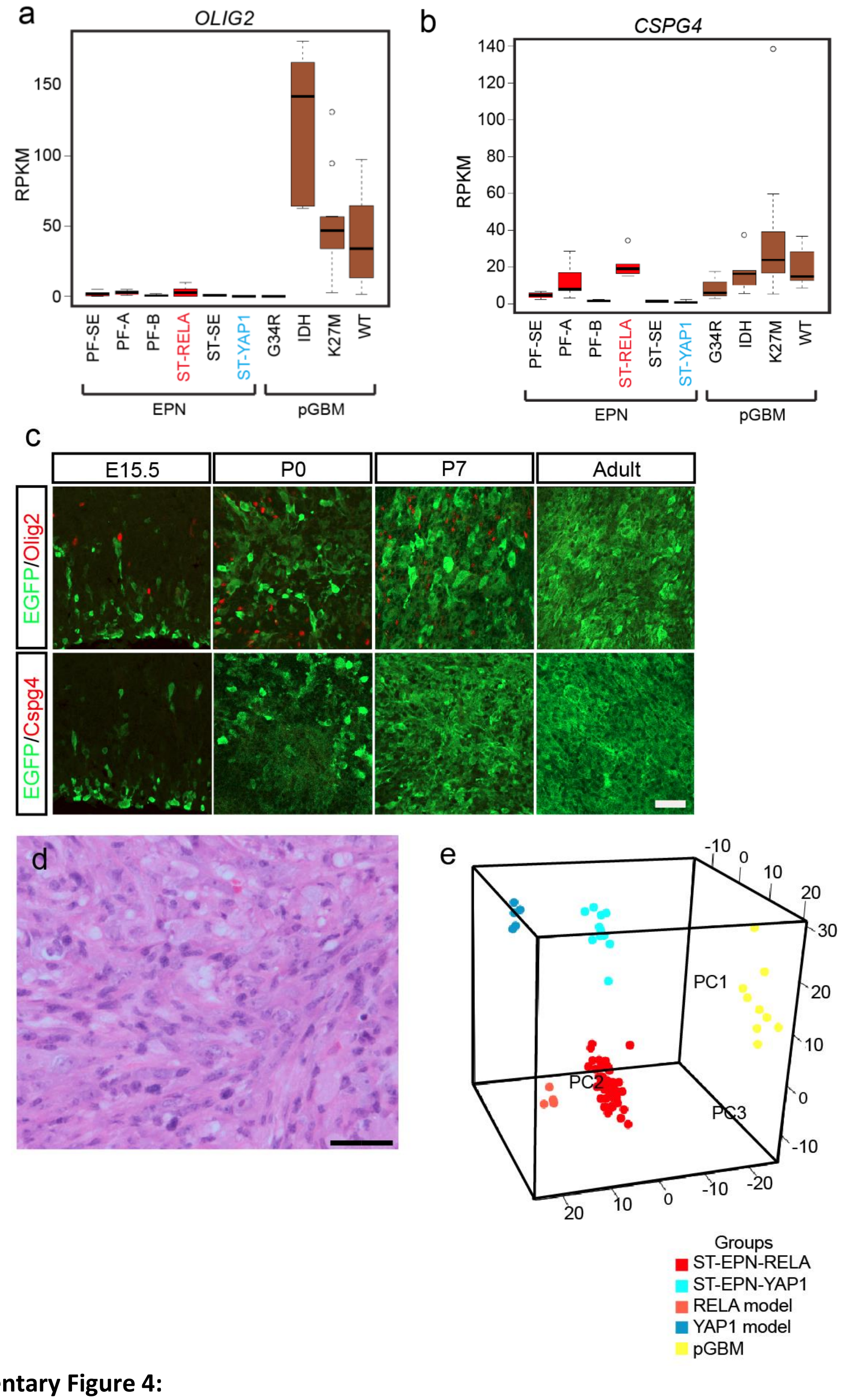

Supplementary Figure 4:

YAP1-MAMLD1-driven transforming cells lack glial progenitor markers in human and mouse.

(a-b) Box plots of transcripts of *OLIG2* (a) and *CSPG4* (b) revealed by RNAseq of human ependymoma (EPN) and pediatric glioblastoma multiforme (pGBM) subgroups. The centre line, box limits, whiskers and points indicate the median, upper/lower quartiles, 1.5x interquartile range and outliers respectively.

(c) IHC of YAP1-MAMLD1-electroporated cells with EGFP (green) and either Olig2 (red, upper panels) or Capg4 (red, lower panels) at indicated stages. The embryos were subjected to in utero electroporation at E13.5. Scale bar, 50 µm.
(d) Histology of a representative YAP1-MAMLD1-driven mouse tumour stained with hematoxylin and eosin. Scale bar, 20 µm.
(e) Principal component analysis of YAP1 mice model (blue) transcriptome profile compared to human ST-EPN-RELA tumours (red) and mice models (coral), ST-EPN-YAP1 (cyan) and pediatric glioblastoma tumours (yellow) based on 308 selected orthologue genes (see Methods).

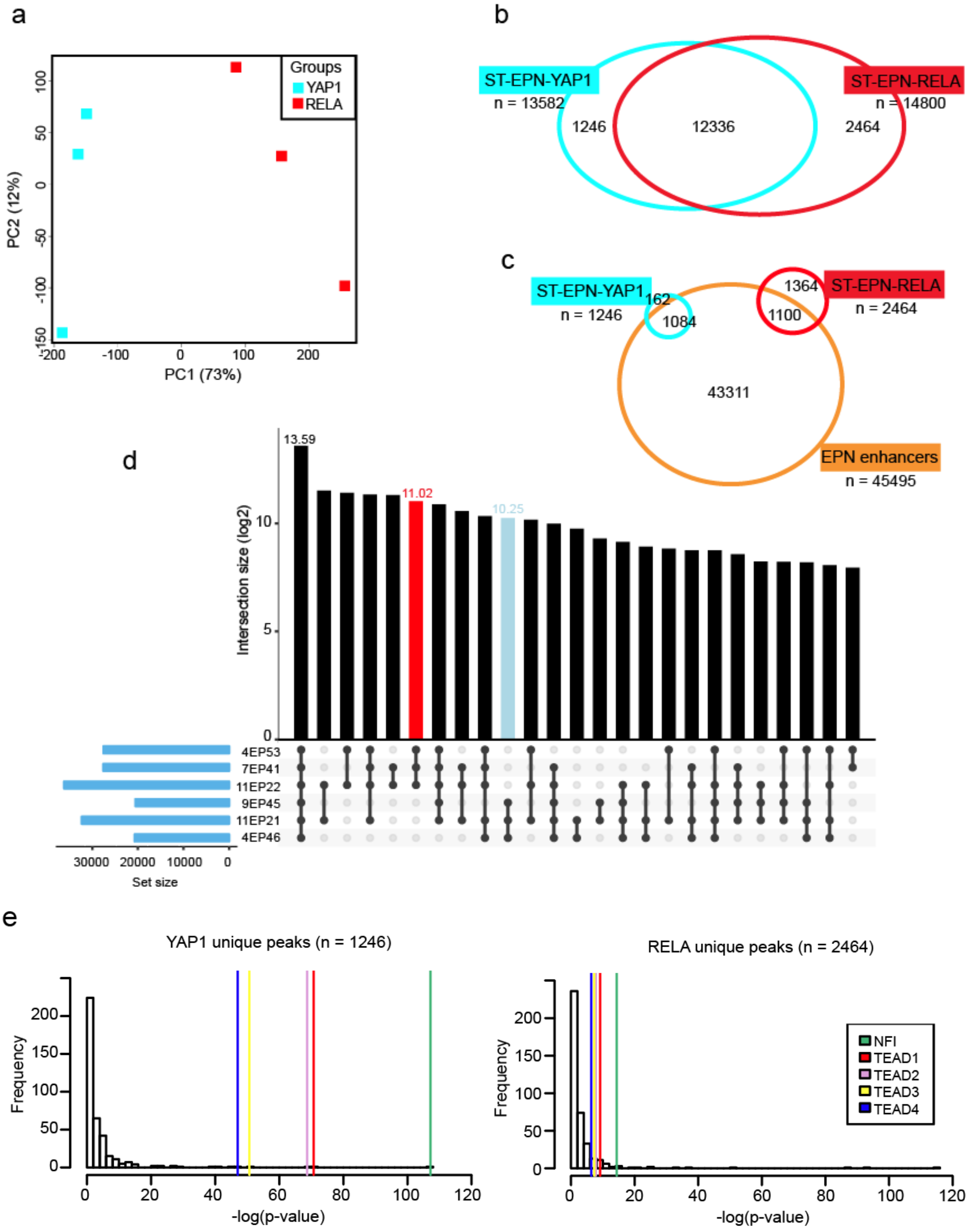
a
Groups
YAP1
RELA
PC2 (12%)
PC1 (73%)
100
50
0
-50
-100
-150
-200
-100
0
100
200
b
ST-EPN-YAP1
n = 13582
ST-EPN-RELA
n = 14800
1246
12336
2464
c
ST-EPN-YAP1
n = 1246
162
1084
1364
ST-EPN-RELA
n = 2464
1100
43311
EPN enhancers
n = 45495
d
13.59
11.02
10.25
Intersection size (log2)
10
5
0
4EP53
7EP41
11EP22
9EP45
11EP21
4EP46
30000
20000
10000
0
Set size
e
YAP1 unique peaks (n = 1246)
RELA unique peaks (n = 2464)
Frequency
200
150
100
50
0
20
40
60
80
100
120
-log(p-value)
NFI
TEAD1
TEAD2
TEAD3
TEAD4

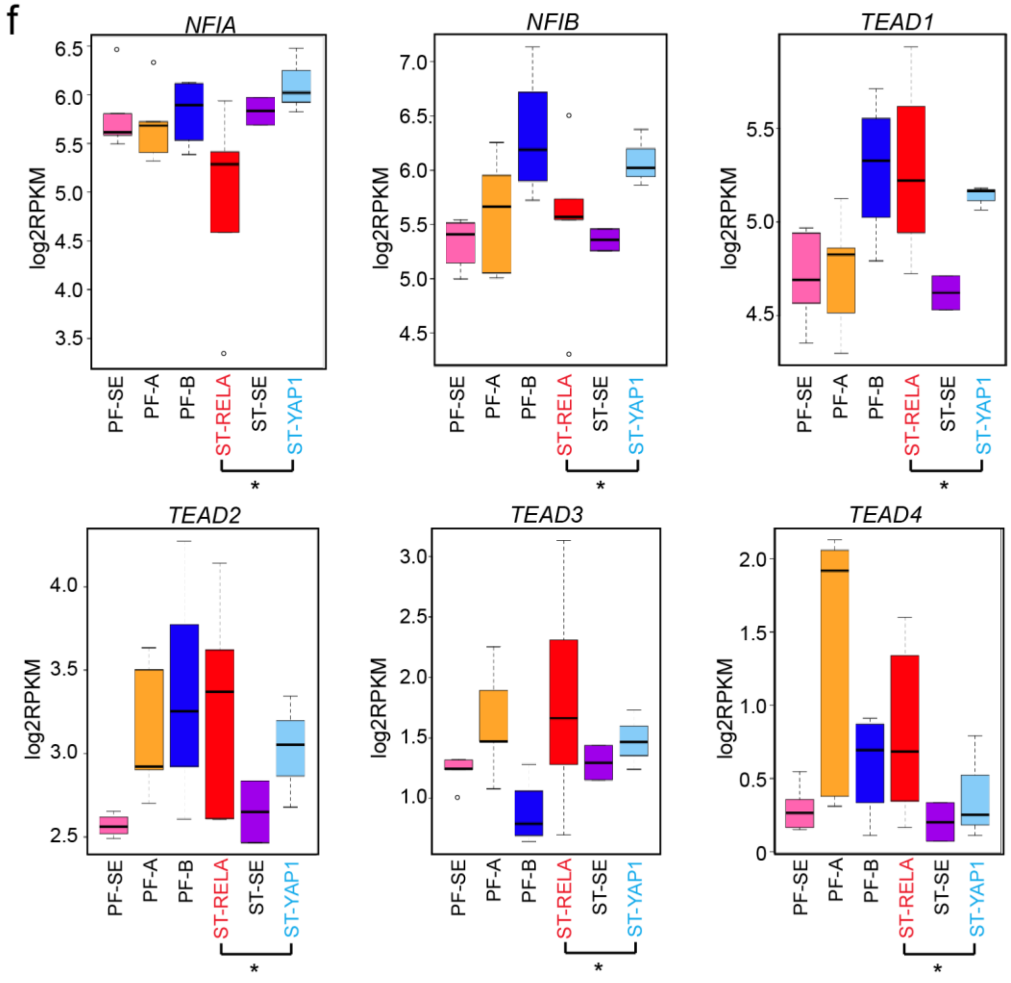

Supplementary Figure 5:

YAP1 ChIP-seq and TEAD motif enrichment in ST-EPN-YAP1 and ST-EPN-RELA tumours.

(a) Principal component analysis of top 5000 variable normalized YAP1 peak signals from ChIP-seq experiments after subtraction of control read counts. YAP1 occupancy is consistent within ST-EPN-YAP1 and ST-EPN-RELA, but significant different between both groups.

(b) Venn diagram displays variance between high-confidence YAP1 peak signals in ST-EPN-YAP1 (cyan blue) and ST-EPN-RELA samples (red).

(c) Venn diagram showing the number of YAP1 peaks of ST-EPN-YAP1 (cyan) and ST-EPN-RELA (red) overlapping with a full enhancer landscape in human EPNs (coral).

(d) Quantitative visualization of set overlaps between YAP1 ChIP-seq peaks across the tumour samples. The highlighted overlap proportions are group specific combined peak selections for ST-EPN-YAP1 (blue) and ST-EPN-RELA (red). (e) Transcription factor motif enrichment in ST-EPN-YAP1 (upper graph) and ST-EPN-RELA (lower graph)-specific YAP1 peaks. Colored lines indicate location of NFI and TEAD1-4 motifs. (f) Box plots presenting comparable expression levels of *NFIA*, *NFIB* and *TEAD1-4* in different molecular subgroups of EPNs. The centre line, box limits, whiskers and points indicate the median, upper/lower quartiles, 1.5x interquartile range and outliers respectively. *p-values = 0.2841 for *NFIA*, 0.7086 for *NFIB*, 0.7322 for *TEAD1*, 0.6084 for *TEAD2*, 0.5391 for *TEAD3* and 0.3435 for *TEAD4* (adjustment computed by Benjamini & Hochberg method).

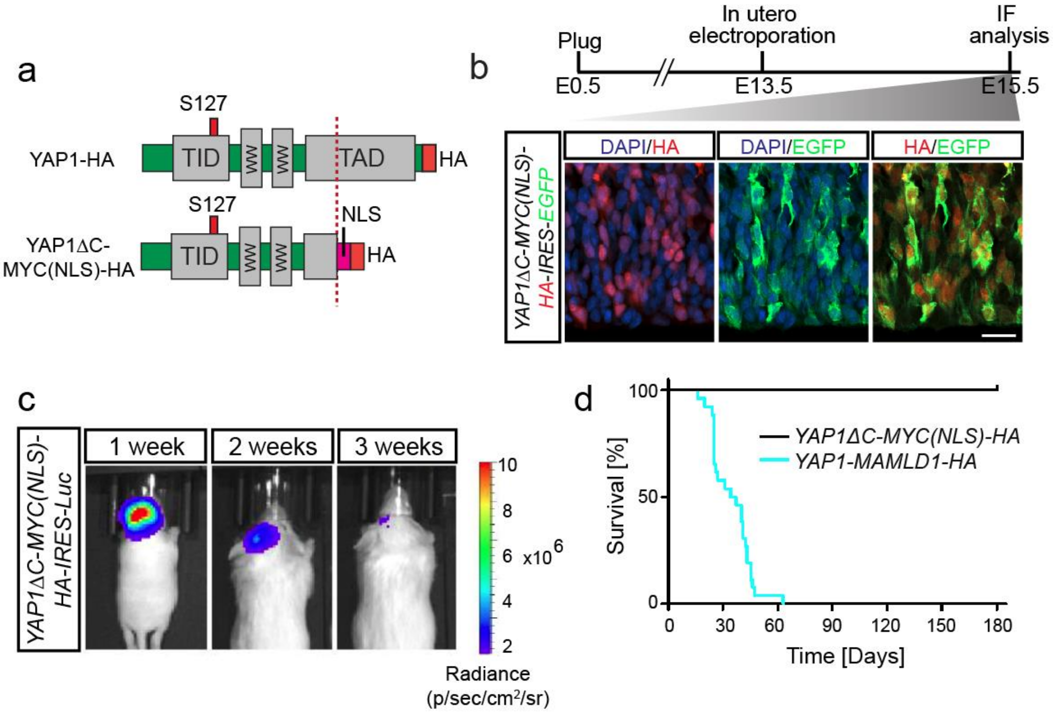

Supplementary Figure 6:

The MAMLD1 domain is required for YAP1-MAMLD1-driven tumourigenesis.

(a) Schematic representation of YAP1ΔC-MYC(NLS)-HA, a putative human MYC-drived nuclear localization signal (NLS) is fused to the C-terminus of the YAP1 domain of YAP1-MAMLD1 (YAP1ΔC).

(b) Immunofluorescence micrographs for subcellular localization of the YAP1-MYC(NLS)-HA protein in the cells of the ventricular zone 2 days after in utero electroporation at E13.5. Double staining was performed for DAPI/HA (left panel) or DAPI/EGFP (mid panel) or EGFP/HA (right panel). Scale bar, 20 µm.

(c, d) Luciferase-based in vivo bioluminescence images (c) and Kaplan-Meier curves (d) for the mice electroporated with *YAP1-MAMLD1-HA-IRES-Luc* (n = 26) and *YAP1ΔC-MYC(NLS)-HA-IRES-Luc* (n = 5). The cyan blue curve for YAP-MAMLD1-HA-Luc is identical to the one shown in Fig. 3k.

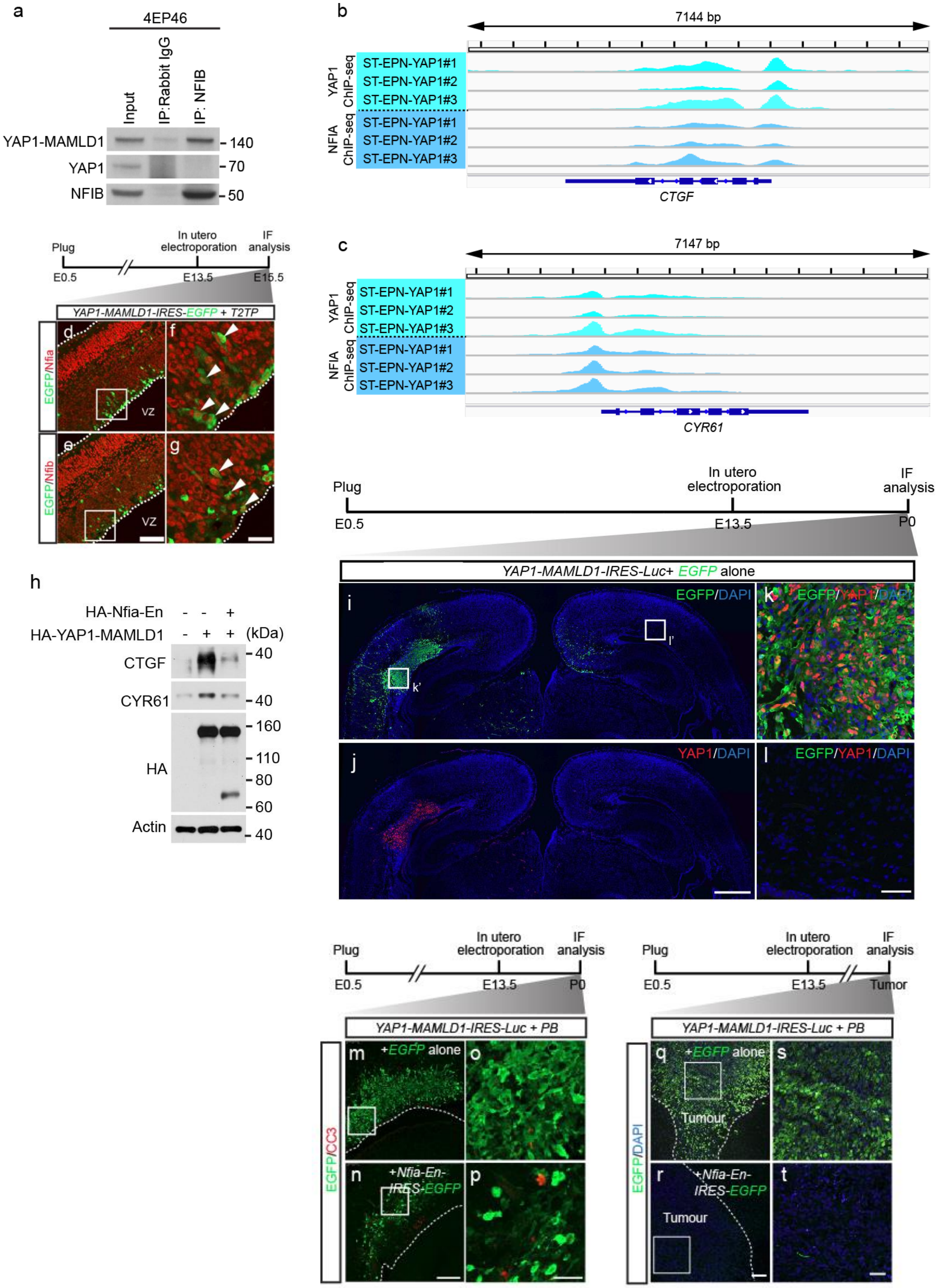

a
4EP46
Input
IP:Rabbit IgG
IP: NFIB
YAP1-MAMLD1
YAP1
NFIB
140
70
50
b
7144 bp
YAP1 ChIP-seq
NFIA ChIP-seq
ST-EPN-YAP1#1
ST-EPN-YAP1#2
ST-EPN-YAP1#3
CTGF
c
7147 bp
CYR61
Plug
In utero electroporation
IF analysis
E0.5
E13.5
E15.5
YAP1-MAMLD1-IRES-EGFP + T2TP
EGFP/Nfia
EGFP/Nfib
VZ
h
HA-Nfia-En
HA-YAP1-MAMLD1
(kDa)
CTGF
CYR61
HA
Actin
40
160
110
80
60
P0
YAP1-MAMLD1-IRES-Luc+ EGFP alone
EGFP/DAPI
EGFP/YAP1/DAPI
YAP1/DAPI
YAP1-MAMLD1-IRES-Luc + PB
+EGFP alone
+Nfia-En-IRES-EGFP
EGFP/CC3
Tumor
Tumour
EGFP/DAPI

Supplementary Figure 7:
NFI proteins interact with the YAP1-MAMLD1 protein via its MAMLD1 domain.
(a) Co-IP with an anti-NFIB antibody on human primary YAP1-MAMLD1 tumour (4EP46).
(b, c) IGV snapshot of ChIP-seq with an anti-NFIA antibody at the loci of *CTGF* and *CYR61*.
(d-g) IHC of E15.5 brains with antibodies against GFP (green) and Nfia (red in d, f) and Nfib (red in e, g) 2 days after electroporation of YAP1-MAMLD1-IRES-EGFP and T2TP at E13.5. (f) and (g) are high magnification views of the area outlined by white squares in (a) and (b), respectively. Scale bar in (e) is 100 μm (for d, e) and the scale bar in (g) is 25 μm (for f, g). Arrowheads indicate electroporated cells positive for Nfia and Nfib.
(h) Western blot of human CTGF and CYR61 in LN229 cells transiently transfected with indicated genes. The transgene protein products were tagged by HA.
(i-l) P0 electroporated brains stained with anti-YAP1 and EGFP antibodies. (k) and (l) are high magnification views of the area outlined by white squares (k’) and (l’) in (i), respectively. Scale bar in (j), 500 μm for (i, j). The scale bar in (l), 50 μm for (k, l).
(m-p) P0 electroporated brains labelled with anti-cleaved caspase-3 (CC3) and EGFP antibodies. (o) and (p) are high magnification views of the area outlined by white squares in (m) and (n), respectively. Scale bar in (n), 150 μm for (m, n). The bar in (p), 40 μm for (o, p). Dotted lined indicate the ventricular zone.
(q-t) IHC of developed tumours with an anti-EGFP antibody. (s) and (t) are high magnification views of the area outlined by white squares in (q) and (r), respectively. Scale bar in (r), 150 μm for (q, r). The bar in (t), 50 μm for (s, t). Dotted lined indicate the boundary between the tumour mass and normal brain tissue.

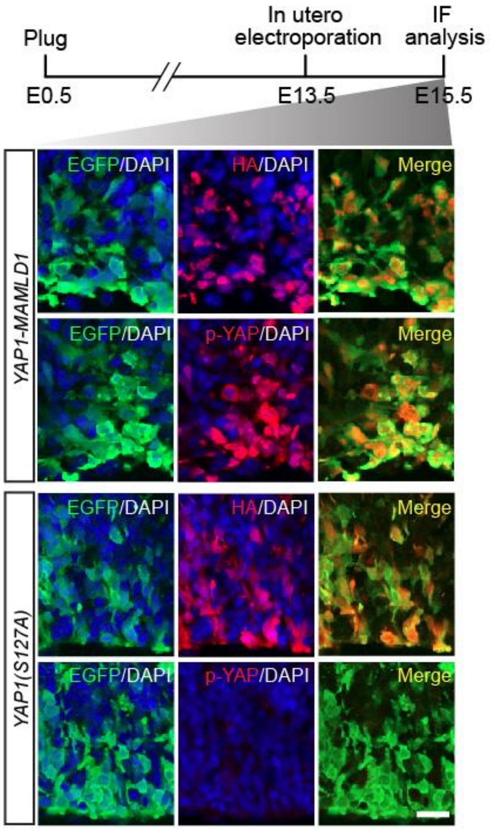

Supplementary Figure 8:
Quality control of the pYAP1 (S127) antibody used in this study
Immunostaining of E15.5 ventricular zone cells in the cerebral cortices electroporated by *YAP1-MAMLD1-HA-IRES-EGFP* (upper panels) and *YAP1(S127A)*-HA-IRES-GFP (lower panels) at E13.5. Scale bar, 20 μm.

For Figure 1g

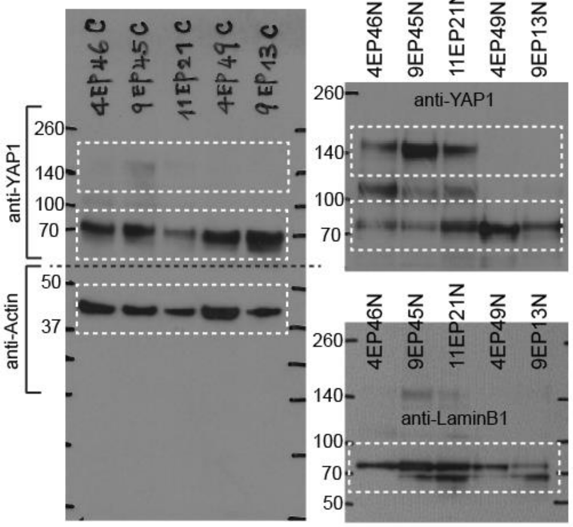

For Figure 1h

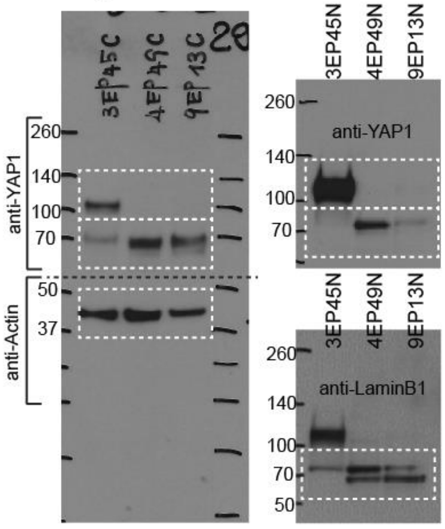

For Figure 1j

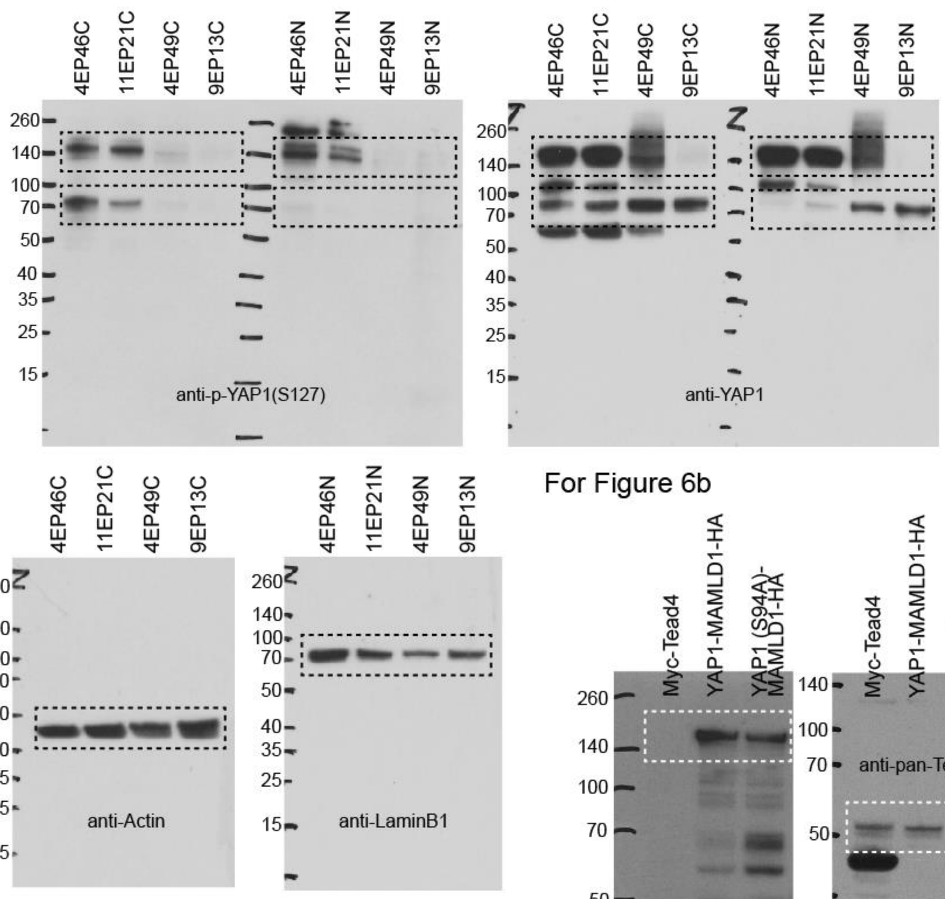

For Figure 6b

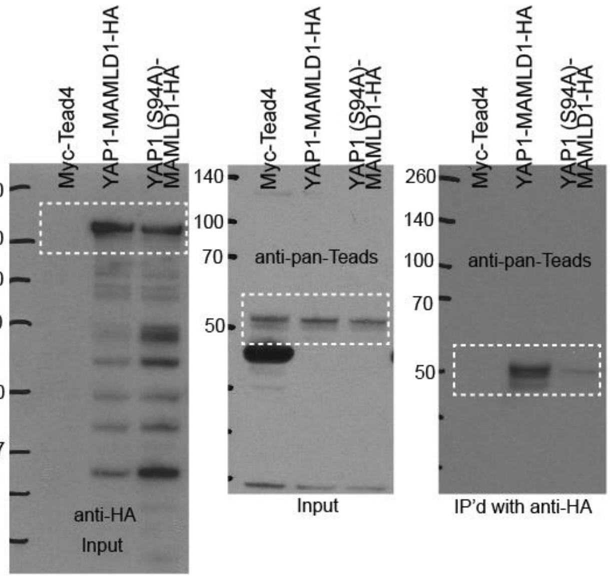

For Figure 2e

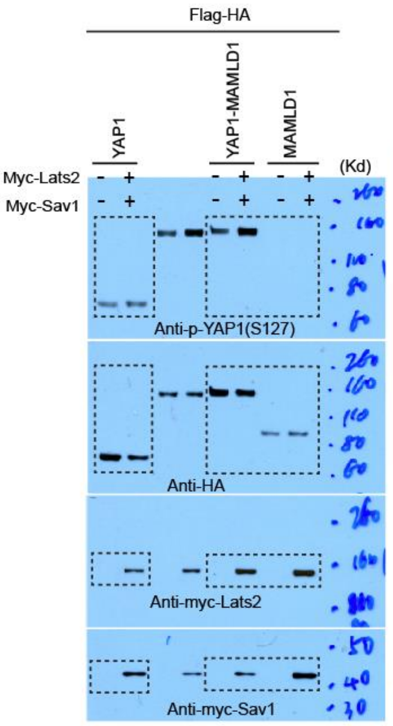

For Figure 2f

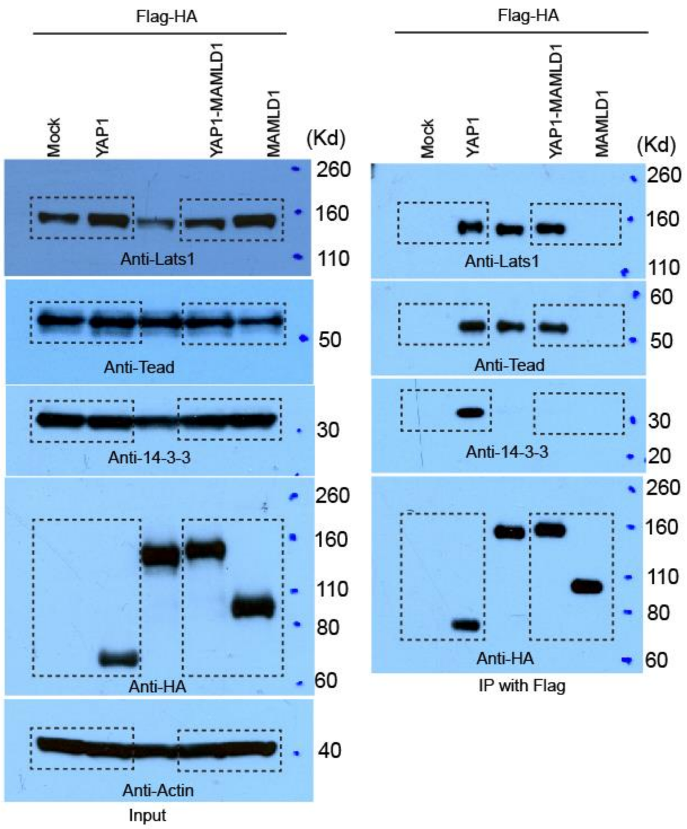

For Figure 6a

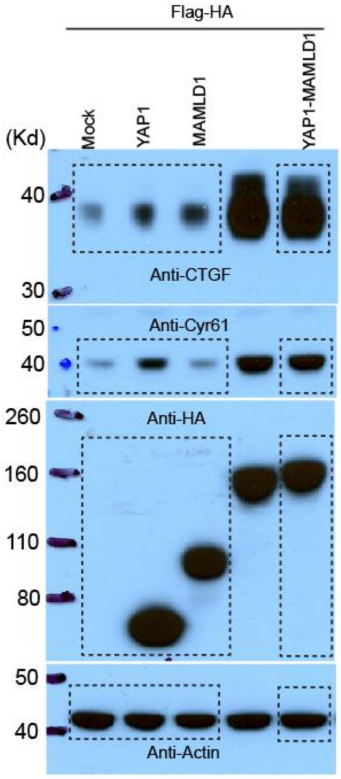

For Figure 6c

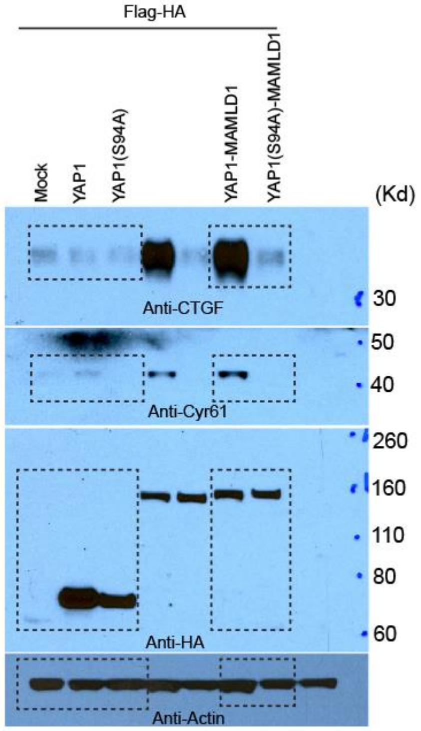

For Figure 7a

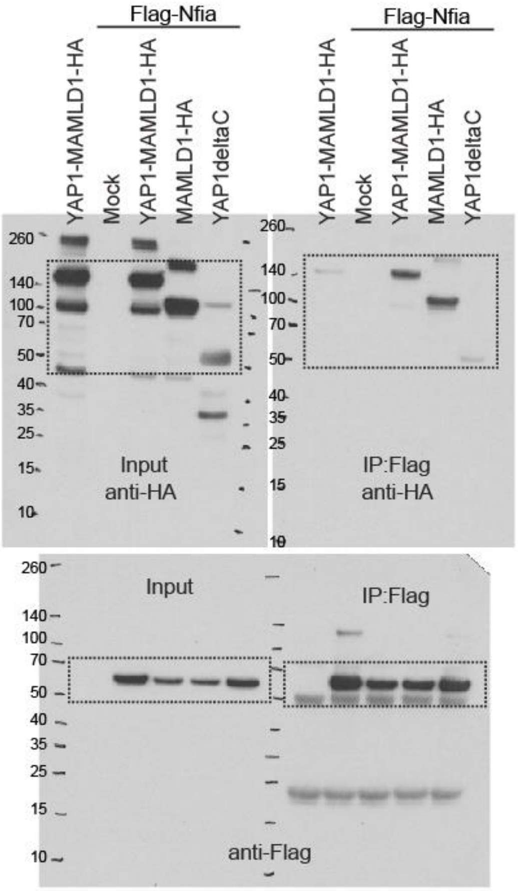

For Figure 7b

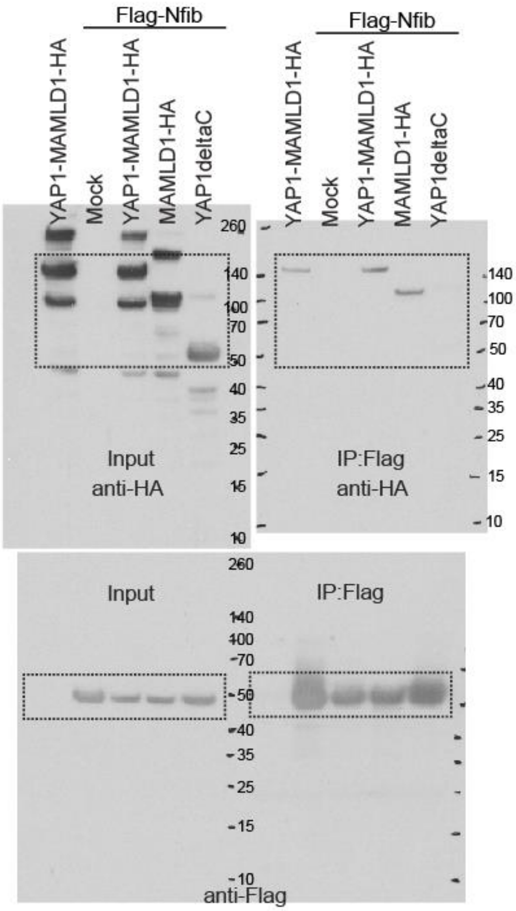

For Supplementary Figure 7a

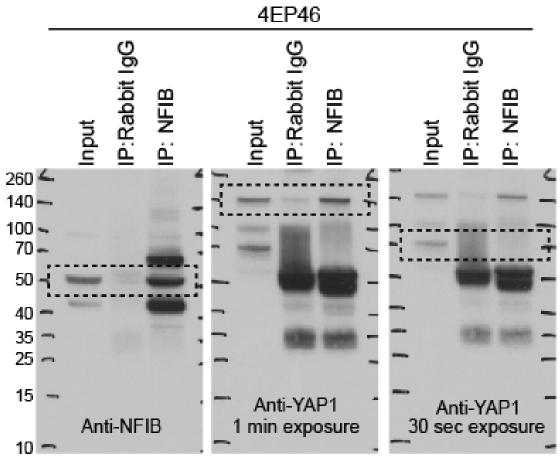

For Supplementary Figure 7h

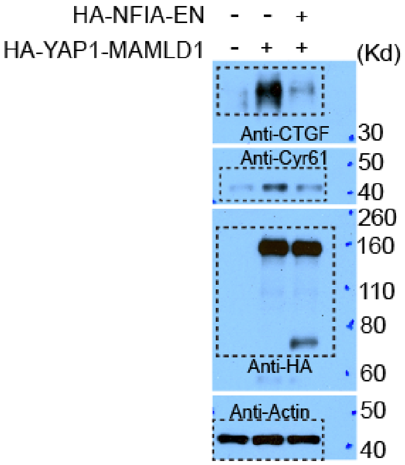

Supplementary Figure. 9: Uncropped data of western blotting in this study.
