# Supplementary material for: YAP1 subgroup supratentorial ependymoma requires TEAD and nuclear factor I-mediated transcriptional programmes for tumorigenesis
Source: Nat Commun. 2019 Sep 2;10:3914. doi: 10.1038/s41467-019-11884-5 (PMC6718408; doi:10.1038/s41467-019-11884-5)
Supplement: Supplementary file 3 — Description of Additional Supplementary Files [file 41467_2019_11884_MOESM3_ESM.pdf]

Description of Additional Supplementary Files

File Name: Supplementary Data 1
Description: The RNA-seq fusion discovery results information and ChIP sequencing QC summary statistics for the study cohort

File Name: Supplementary Data 2
Description: Transcription factor binding motif enrichment in ST-EPNYAP1-specific YAP1 peaks, ST-EPN-RELA-specific YAP1 peaks and common between ST-EPN-RELA and ST-EPN-YAP1.

File Name: Supplementary Data 3
Description: List of genes associated with ST-EPN-YAP1-specific regulatory elements that overlap with ST-EPN-YAP1-specific and ST-EPN-RELAspecific YAP1 peaks Supplementary Data

File Name: Supplementary Data 4
Description: GO analysis of genes associated with ST-EPN-YAP1- specific regulatory elements that overlap with ST-EPN-YAP1-specific YAP1 peaks

File Name: Supplementary Data 5
Description: Transcription factor binding motif enrichment in ST-EPNYAP1-specific and ST-EPN-RELA-specific YAP1 peaks overlapping with group specific and common enhancers/superenhancers

File Name: Supplementary Data 6
Description: List of confident differentially expressed genes between YAP1 and RELA mouse models confirmed in human tumour comparison
