# Supplementary material for: YAP1 subgroup supratentorial ependymoma requires TEAD and nuclear factor I-mediated transcriptional programmes for tumorigenesis
Source: Nat Commun. 2019 Sep 2;10:3914. doi: 10.1038/s41467-019-11884-5 (PMC6718408; doi:10.1038/s41467-019-11884-5)
Supplement: Supplementary file 10 — Reporting Summary [file 41467_2019_11884_MOESM10_ESM.pdf]

nature research

Corresponding author(s): Wei Li, Daisuke Kawauchi

Last updated by author(s): Jan 18, 2019

Reporting Summary

Nature Research wishes to improve the reproducibility of the work that we publish. This form provides structure for consistency and transparency in reporting. For further information on Nature Research policies, see Authors & Referees and the Editorial Policy Checklist .

Statistics

For all statistical analyses, confirm that the following items are present in the figure legend, table legend, main text, or Methods section.

n/a	Confirmed	
☐	☒	The exact sample size (*n*) for each experimental group/condition, given as a discrete number and unit of measurement
☐	☒	A statement on whether measurements were taken from distinct samples or whether the same sample was measured repeatedly
☐	☐	The statistical test(s) used AND whether they are one- or two-sided<br>*Only common tests should be described solely by name; describe more complex techniques in the Methods section.*
☐	☒	A description of all covariates tested
☐	☒	A description of any assumptions or corrections, such as tests of normality and adjustment for multiple comparisons
☐	☒	A full description of the statistical parameters including central tendency (e.g. means) or other basic estimates (e.g. regression coefficient) AND variation (e.g. standard deviation) or associated estimates of uncertainty (e.g. confidence intervals)
☐	☒	For null hypothesis testing, the test statistic (e.g. *F*, *t*, *r*) with confidence intervals, effect sizes, degrees of freedom and *P* value noted<br>*Give P values as exact values whenever suitable.*
☒	☐	For Bayesian analysis, information on the choice of priors and Markov chain Monte Carlo settings
☒	☐	For hierarchical and complex designs, identification of the appropriate level for tests and full reporting of outcomes
☒	☐	Estimates of effect sizes (e.g. Cohen's *d*, Pearson's *r*), indicating how they were calculated

Our web collection on statistics for biologists contains articles on many of the points above.

Software and code

Policy information about availability of computer code

Data collection	All the sequencing data was generated in DKFZ Heidelberg. No software was used to collect additional data.
Data analysis	The sequencing data analysis was performed based on the usage of open-source software. For ChIP-seq data the reads alignment was performed with BWA v0.5.10, peak calling with MACS v1.4, transcription factor motif enrichment analysis with HOMER v4.8.3. RNA-seq reads alignment was performed with STAR 2.3.0, gene counts computed with Subread v1.4.6, fusion calling performed with InFusion v0.6.3. Affymetrix data processing was performed with R packages Affy 1.52.0 and Limma v3.30.13.

For manuscripts utilizing custom algorithms or software that are central to the research but not yet described in published literature, software must be made available to editors/reviewers. We strongly encourage code deposition in a community repository (e.g. GitHub). See the Nature Research guidelines for submitting code & software for further information.

Data

Policy information about availability of data

All manuscripts must include a data availability statement. This statement should provide the following information, where applicable:

- Accession codes, unique identifiers, or web links for publicly available datasets
- A list of figures that have associated raw data
- A description of any restrictions on data availability

All described sequencing data from ependymoma tumours (RNA-seq; YAP1, NFIA and H3K27ac ChIP-seq) is available from EGA under the accession number EGAS00001002696 [https://www.ebi.ac.uk/ega/studies/EGAS00001002696]. The 450K-methylation arrays for ependymoma tumours are available from GEO under ID GSE65362 [https://www.ncbi.nlm.nih.gov/geo/query/acc.cgi?acc=GSE65362]. The Affymetrix expression datasets are available under the following IDs from GEO: for ependymoma tumours - GSE64415 [https://www.ncbi.nlm.nih.gov/geo/query/acc.cgi?acc=GSE64415], for glioblastoma tumours – GSE134404 [https://www.ncbi.nlm.nih.gov/geo/query/acc.cgi?acc=GSE134404], for YAP1 and RELA mouse models – GSE110625 [https://www.ncbi.nlm.nih.gov/geo/query/acc.cgi?acc=GSE110625].

Field-specific reporting

Please select the one below that is the best fit for your research. If you are not sure, read the appropriate sections before making your selection.

☒ Life sciences ☐ Behavioural & social sciences ☐ Ecological, evolutionary & environmental sciences

For a reference copy of the document with all sections, see nature.com/documents/nr-reporting-summary-flat.pdf

Life sciences study design

All studies must disclose on these points even when the disclosure is negative.

Sample size	No sample size calculation was performed.<br>The Methods section and Legends contain a description of the sample size.
Data exclusions	No data points were excluded in our study.
Replication	All attempts at replication were successful.
Randomization	No randomization was done in our animal experiments. All mice used in this study have the same genetic background (CD-1).
Blinding	Double-blinded was done in the quantifications in Figure 3 and 7.

Reporting for specific materials, systems and methods

We require information from authors about some types of materials, experimental systems and methods used in many studies. Here, indicate whether each material, system or method listed is relevant to your study. If you are not sure if a list item applies to your research, read the appropriate section before selecting a response.

Materials & experimental systems

n/a	Involved in the study	
☐	☒	Antibodies
☐	☒	Eukaryotic cell lines
☒	☐	Palaeontology
☐	☒	Animals and other organisms
☒	☐	Human research participants
☒	☐	Clinical data

Methods

n/a	Involved in the study	
☐	☒	ChIP-seq
☒	☐	Flow cytometry
☒	☐	MRI-based neuroimaging

Antibodies

Antibodies used	The information about the antibodies used in this study is described in the Methods.<br>YAP1 (Cat#GTX129151, GeneTex, Lot#41500), YAP1 (Cat#14074, Cell signaling, Lot#1), YAP1 (Cat#sc-15407X, SCBT, Lot#D2115), p-YAP(S127) (Cat#ab76252, abcam, Lot#GR196211-35), p-YAP(S127) (Cat#13008, Cell signaling, Cat#1), Ki67 (Cat#ab15580, Abcam, Lot#GR3198167-1), HA (Cat#3724, CST), HA (Cat#MMS-101P, BioLegend), Myc (Clone#9E10, Developmental Studies Hybridoma Bank), Lats1 (Cat#3447, CST), Actin (Cat#A5316, Sigma-Aldrich, Cat#ab49900, abcam), CTGF (Cat#sc-14939, SCBT), Cyr61 (Cat#sc-13100, SCBT), 14-3-3 (Cat#sc-629, SCBT), FLAG (Clone#M2, Sigma, Lot#SLBW5142), Pan-Tead (Cat#13295, Cell signaling, Lot#1), TBR2/Eomes (Cat#ab23345, Abcam), NG2 (Cat#ab129051, Abcam, Lot#GR3205582-1), PAX6 (Cat#PPRB-278P, Covance, Lot#B244513), Olig2 (Cat#AB9610, Millipore), NFIA (Cat#HPA008884, Sigma, Lot#A104295), NFIB (Cat#HPA003956, Sigma, Lot#A114436) Cleaved Caspase 3 (Cat#9664, CST) and GFP (#ab13970, Abcam, Cat#GR3190550-3).
Validation	The following antibodies were validated in our and other previous studies:<br>YAP1 (SCBT): https://www.nature.com/articles/ncomms10498<br>Ki67 (Abcam): https://www.nature.com/articles/ncomms14758<br>PAX6 (Covance): https://www.nature.com/articles/ncomms14758<br>GFP (Abcam): https://www.nature.com/articles/ncomms14758<br>FLAG (M2, Sigma):https://www.nature.com/articles/ncomms14758<br>TBR2/Eomes (Abcam): https://www.nature.com/articles/nature16546<br>Pan-Tead (Cell signaling): https://www.sciencedirect.com/science/article/pii/S0960982218315963<br><br>The following antibodies were quality-checked in the manufacture's websites:<br>YAP1 (GeneTex): http://www.genetex.com/YAP1-antibody-GTX129151.html<br>YAP1 (CST): https://www.cellsignal.de/products/primary-antibodies/yap-d8h1x-xp-rabbit-mab/14074<br>p-YAP(S127) (abcam): https://www.abcam.com/yap1-phospho-s127-antibody-ep1675y-ab76252.html

p-YAP(S127) (CST): https://www.cellsignal.de/products/primary-antibodies/phospho-yap-ser127-d9w2i-rabbit-mab/13008, Supplementary Fig. 8 in this study
CTGF (SCBT): https://www.scbt.com/scbt/product/ctgf-antibody-l-20
Cyr61 (SCBT): https://www.scbt.com/scbt/product/cyr61-antibody-h-78?requestFrom=search
HA (CST): https://www.cellsignal.de/products/primary-antibodies/ha-tag-c29f4-rabbit-mab/3724
Myc (9E10, Developmental Studies Hybridoma Bank)
NG2 (Abcam): https://www.abcam.com/ng2-antibody-ab129051.html
Olig2 (Millipore):http://www.merckmillipore.com/DE/de/product/Anti-Olig-2-Antibody,MM_NF-AB9610
NFIA (Sigma):https://www.proteinatlas.org/ENSG00000162599-NFIA/tissue
NFIB (Sigma):https://www.proteinatlas.org/ENSG00000147862-NFIB/antibody

Eukaryotic cell lines

Policy information about cell lines

Cell line source(s)	ATCC (for HEK293T, NIH/3T3, LN229)
Authentication	None of the cell lines used were authenticated.
Mycoplasma contamination	All cell lines were tested negative for mycoplasma contamination.
Commonly misidentified lines (See ICLAC register)	N/A

Animals and other organisms

Policy information about studies involving animals; ARRIVE guidelines recommended for reporting animal research

Laboratory animals	CD1, male and female mice
Wild animals	This study did not involve wild animals.
Field-collected samples	This study did not involve samples collected from the field.
Ethics oversight	All animal experiments for this study were conducted according to the Penn State University Institutional Animal Care and Use Committee and the animal welfare regulations approved by the Animal Care and Use Committee of the National Institute of Neuroscience, NCNP, Japan and the responsible authorities in Germany (Regierungspräsidium Karlsruhe, approval number: G204/16).

Note that full information on the approval of the study protocol must also be provided in the manuscript.

ChIP-seq

Data deposition

- [x] Confirm that both raw and final processed data have been deposited in a public database such as GEO.
- [ ] Confirm that you have deposited or provided access to graph files (e.g. BED files) for the called peaks.

Data access links<br>*May remain private before publication.*	https://www.ebi.ac.uk/ega/studies/EGAS00001002696
Files in database submission	YAP1 ChIP-seq data:<br>4EP46 AS-174271-LR-26523_R1/R2.fastq.gz<br>4EP53 AS-174282-LR-26524_R1/R2.fastq.gz<br>7EP41 AS-174280-LR-26524_R1/R2.fastq.gz<br>9EP45 AS-174278-LR-26524_R1/R2.fastq.gz<br>11EP21 AS-174276-LR-26524_R1/R2.fastq.gz<br>11EP22 AS-174273-LR-26523_R1/R2.fastq.gz<br>NFIA ChIP-seq data:<br>4EP46: AS-338689-LR-42012_R1/R2.fastq.gz<br>9EP45: AS-338687-LR-42012_R1/R2.fastq.gz<br>11EP21 AS-338685-LR-42012_R1/R2.fastq.gz
Genome browser session (e.g. UCSC)	Full prepared IGV session can be accessed based on a request.

Methodology

Replicates	Each tumor subgroup (ST-EPN-RELA/ST-EPN-YAP1) was represented by 3 unique samples.

Sequencing depth	The sequencing procedure for 6 YAP1 and 3 NFIA ChIP tumor samples was performed with Illumina HiSeq 2000 (read size 125 bp) and resulted in 1128693848 paired-end reads in total (~125410428 mean reads per sample). From them, ~91% reads were mapped with formation of correct pairs to hg19 reference. Further, in order to prepare for the peaks calling procedure, duplicates removal was performed for each sample alignment using samtools v0.1.18.
Antibodies	YAP1 antibody (Cat#sc-15407X, SCBT, Lot#D2115) and NFIA antibody (Cat#HPA008884, Sigma, Lot#A104295)
Peak calling parameters	Peaks computation was performed with MACS v1.4 tool using whole genome sequencing data from the same samples as background control and filtered with min p-value limit 1e-9.
Data quality	The quality control was performed with Qualimap v2.2.1 tool mode BAM QC applied for each alignment file to measure mapped reads proportion, coverage and insert size. The peaks computation resulted in ~43935 mean peaks per sample.
Software	The peaks per group were combined using Bedtools 2.24.0 merge command. Selection of group specific peaks and combination with enhancers was performed based on overlaps computation using GenomicRanges 1.26.4 R package. Motif enrichment analysis was performed using HOMER v4.8.3 with integration of TEAD3 motif from JASPAR database. Visualization was performed in IGV.
